# 吉非替尼联合心包灌注治疗晚期非小细胞肺癌的疗效观察

**DOI:** 10.3779/j.issn.1009-3419.2018.01.10

**Published:** 2018-01-20

**Authors:** 晓朦 王, 槿 陈, 佳琪 姚, 人花 郭

**Affiliations:** 1 210029 南京，南京医科大学第一附属医院肿瘤科 Department of Oncology, First Affiliated Hospital of Nanjing Medical University, Nanjing 210029, China; 2 211166 南京，南京医科大学 Nanjing Medical University, Nanjing 211166, China

**Keywords:** 恶性心包积液, 吉非替尼, 羟基喜树碱, 肺肿瘤, Malignant pericardialeffusion, Gefitinib, Hydroxycamptothecine, Lung neoplasms

## Abstract

**背景与目的:**

表皮生长因子受体（epidermal growth factor receptor, *EGFR*）突变的非小细胞肺癌（non-small cell lung cancer, NSCLC）是肺癌的一个重要亚型。*EGFR*突变的NSCLC患者合并恶性心包积液的发生率较高，但目前针对此型肺癌患者治疗方案的研究较少。

**方法:**

本研究对我院2010年1月-2016年12月期间确诊的*EGFR*敏感突变的NSCLC合并恶性心包积液患者的临床资料和治疗情况进行回顾性分析，具体分为以下三组治疗模式：口服吉非替尼联合心包灌注羟基喜树碱组（吉非替尼/HCPT），静脉化疗联合心包灌注羟基喜树碱组（化疗/HCPT）及吉非替尼单药组，探讨不同治疗模式患者病情的转归。

**结果:**

273例晚期*EGFR*敏感突变的NSCLC患者中，29例初诊时有心包积液，剔除6例少量心包积液无法取得细胞学分析的患者，共23例纳入分析。患者总中位心包内疾病无进展时间（progression-free survival, PFS）为247天；吉非替尼/HCPT组患者PFS（460天）优于化疗/HCPT组患者（94天），（*P*=0.008）；吉非替尼/HCPT组患者的PFS优于吉非替尼单药组患者（131天），（*P*=0.032）。同时对肺部原发灶的治疗分析显示：吉非替尼/HCPT组患者疗效优于化疗/HCPT组患者（客观有效率：33.3% *vs* 12.5%；疾病控制率：86.7% *vs* 62.5%）；吉非替尼/HCPT组与吉非替尼单药组的患者，肺部肿瘤疗效无差异。且三组均未观察到明显的不良反应。

**结论:**

一线吉非替尼联合心包灌注羟基喜树碱治疗有助于延长*EGFR*突变合并恶性心包积液的晚期NSCLC患者心包内PFS，且患者耐受性好，无明显不良反应。因例数少，尚有待更多样本多中心前瞻性临床研究证实。

## Abstract (en)

**Background and objective:**

Epidermal growth factor receptor (*EGFR*) mutation non-small cell lung cancer (NSCLC) is an important subtype of lung cancer. The incidence of malignant pericardial effusion (MPCE) in *EGFR*-mutant NSCLC patients is high. However, there are few researches on the treatmentof this type of patients.

**Methods:**

We collected data on clinical characteristics and treatment of advanced NSCLC patients who harboring *EGFR* mutants and MPCE between January 2010 and December 2016. The treatments were divided into three groups: oral gefitinib combined with pericardial perfusion of hydroxycamptotheci (HCPT) group (gefitinib/HCPT); intravenous chemotherapy combined with pericardial perfusion of HCPT group (chemotherapy/HCPT) and gefitinib monotherapy group. And we retrospectively analyzed patients' outcomes in three groups.

**Results:**

In 273 advanced NSCLC patients with *EGFR* mutations, 29 cases had pericardial effusion, among which 6 patients with small amount of pericardial effusion were excluded, and 23 patients were analyzed. Median pericardium progression free survival (PFS) was 247 days. PFS for gefitinib/HCPT group (460 days) was superior to PFS for chemotherapy/HCPT group (94 days, *P*=0.008) and gefitinib monotherapy group (131 days, *P*=0.032). As for the efficacy of primary pulmonary lesions, the efficacy in gefitinib/ HCPT group was superior to chemotherapy/HCPT group [objective response rate (ORR): 33.3% *vs* 12.5%; disease control rate (DCR): 86.7% *vs* 62.5%]. There is no difference of ORR and DCR between gefitinib/HCPT group and gefitinib monotherapy group. No obvious adverse reaction was observed in all three groups.

**Conclusion:**

First-line gefitinib therapy combined with pericardial perfusion of HCPT can improve pericardium PFS for advanced NSCLC patients who harboring *EGFR* mutants andmalignantpericardial effusion. This finding should be confirmed further through multicenter, prospective clinical trials with large sample size.

在世界范围内，肺癌的发病率和死亡率均居恶性肿瘤的前列。非小细胞肺癌（non-small cell lung cancer, NSCLC）约占所有肺癌病例的85%^[1]^，其中表皮生长因子受体（epidermal growth factor receptor, *EGFR*）敏感突变的NSCLC是肺癌的一个重要亚型。随着基础研究及药物研发的进展，近10年来，靶向治疗已成为晚期NSCLC的重要治疗模式之一。对于晚期NSCLC合并*EGFR*突变的患者，EGFR酪氨酸激酶抑制剂（EGFR tyrosine kinase inhibitors, EGFR-TKIs）是首选的治疗方案，如吉非替尼和厄洛替尼能明显改善这类患者的疗效和生活质量^[2, 3]^。

心包积液（pericardial effusion）大多是由恶性肿瘤转移所致，肺癌是这些恶性肿瘤中最常见的类型，占到28%-31%^[4-6]^。恶性心包积液（malignant pericardial effusion, MPCE）可导致心包填塞和难治性心力衰竭，并影响患者呼吸、循环系统的功能和生活质量。同时，有MPCE的晚期NSCLC患者的预后很差，是晚期肺癌死亡的一个重要原因。

既往研究表明，心包穿刺和心包腔灌注药物是对合并MPCE的恶性癌症的首选治疗方法。心包腔穿刺可引流心包内积液，引流后心包腔药物灌注治疗可起到局部抗肿瘤治疗且防止心包内积液复发的作用。这是一种有效、方便、安全的治疗措施。顺铂作为常见的抗肿瘤化疗药，通过心包内灌注的方法广泛地应用于治疗肺癌合并的MPCE^[7, 8]^，但因其严重的消化道反应、心肌损伤或心律失常等不良反应，往往导致患者无法耐受^[9]^。因此在临床工作中我们迫切需要寻找高效低毒的心包腔灌注药物。羟基喜树碱（hydroxycamptothecine, HCPT）对许多恶性肿瘤有很好的疗效，如胃癌、结肠癌、头颈部肿瘤和肝癌^[10-13]^。既往研究^[14, 15]^表明，胸腹腔灌注HCPT是治疗胸腹水比较有效的方法，且未观察到明显的不良反应。

因此我们收集了我院肿瘤科近年来确诊*EGFR*突变合并MPCE的晚期NSCLC患者的临床资料，对他们的治疗情况进行回顾性分析，了解*EGFR*突变合并MPCE的NSCLC患者的治疗状况、MPCE的控制情况及生存情况，为临床医生提供治疗思路。

## 1 资料与方法

### 1.1 入组患者

选择2010年1月1日-2016年12月31日期间南京医科大学第一家附属医院肿瘤科收治的644例晚期NSCLC患者。入选标准：①初诊有*EGFR*敏感突变:18、19或21外显子突变。患者的EGFR突变类型的检测均由我院病理科运用扩增阻滞突变系统（Amplification Refractory Mutation System, ARMS）法进行检测。②中度至大量的MPCE，经计算机断层扫描（computed tomography, CT）、心超或核磁共振成像证实，且经病理学或细胞学诊断证实为肺癌细胞。MPCE的评价标准：按Weitzman等^[16]^提出的方法划分心包腔内最大舒张期暗区 < 10 mm时为少量积液，10 mm-19 mm为中量积液， > 20 mm为大量积液，本次研究中入选患者均合并中到大量MPCE。

记录患者的临床资料，包括性别、年龄、吸烟状况、美国东部协作肿瘤组体力活动状态（Eastern Cooperative Oncology Group performance status, ECOG PS）评分、分期、组织学类型、治疗情况及转归等。肿瘤-淋巴结-转移（tumor-node-metastasis, TNM）分期以美国癌症联合会（American Joint Commitee for Cancer, AJCC）第七版分期系统为标准。本研究涉及的所有步骤均符合南京医科大学第一附属医院机构研究委员会的伦理标准，并符合1964年《赫尔辛基宣言》及其后修正案。对于这种类型的研究（回顾性数据分析），不需要签署正式的患者知情同意书。

### 1.2 治疗方案

具体分三组：①吉非替尼/HCPT组（9例）：口服吉非替尼（250 mg，每日一次，阿斯利康公司），心包灌注羟基喜树碱（10 mg/次，3周一次）；②化疗/HCPT组（8例）：静脉滴注培美曲塞（500 mg/m^2^，第一天），或多西他赛（75 mg/m^2^，第一天），联合卡铂（AUC4-6，第一天）化疗，21天为1疗程，心包灌注羟基喜树碱（10 mg/次，3周一次）；③吉非替尼单药组（6例）：口服吉非替尼（250 mg，每天一次）。

如有明显药物相关不良反应导致患者无法耐受，可酌情减量。化疗失败患者二线均接受吉非替尼治疗。

其中心包腔穿刺的方法为：经B超定位后穿刺点取左侧剑肋角下方。患者取端坐位或半卧位，常规消毒皮肤，铺无菌洞巾，以2%利多卡因行穿刺点局部浸润麻醉。进针同时回抽，抽出积液证实进入心包腔后，经穿刺针尾端缓慢植入导丝，拔穿刺针，扩张管沿导丝扩张皮肤及皮下组织，中心静脉导管沿导丝进入心包腔内10 cm-15 cm，退出导丝，固定中心静脉导管。外接无菌引流袋缓慢引流，胶布固定导管。

### 1.3 疗效评价

心包积液：客观疗效判断采用世界卫生组织制定的浆膜腔积液疗效评价标准（Response Evaluation Criteria in Solid Tumors, RECIST）1.1版非靶病灶评价标准^[17]^，具体如下：完全缓解（complete response, CR），心包积液完全消失，至少维持4周；部分缓解（partial response, PR），心包积液量减少≥50%，至少维持4周；无效（no response, NR），心包积液无变化，或积液量减少 < 50%。心包腔灌注后2周内复查心脏彩超，与治疗前对比评价疗效。4周后再次复查确认疗效。

实体瘤：疗效评价采用RECIST 1.1版^[18]^。疗效判定包括CR、PR、疾病稳定（stable disease, SD）及疾病进展（progressive disease, PD）。客观缓解率（objective response rate, ORR）包括CR和PR，疾病控制率（disease control rate, DCR）包括CR、PR和SD。初始治疗1个月后进行肺部病灶的疗效评价，之后每2个月随访一次。

### 1.4 不良反应的评估

评估患者的血液系统不良反应（白细胞减少症、贫血和血小板减少症等）、非血液系统不良反应（皮疹、肝肾功能障碍和腹泻等）。所有的不良反应都是根据国家癌症研究所的常见毒性标准3.0（National Cancer Institute Common Toxicity Criteria version 3.0, CTC 3.0）^[19]^来评估的。

### 1.5 统计学方法

心包内无进展生存时间（progression free survival, PFS）为一线治疗开始至MPCE进展或死亡的时间，全组随访日期截止至2016年12月31日。采用统计软件SPSS 24.0进行数据分析。PFS采用*Kaplan-Meier*法计算，组间差异采用*Log-rank*分析。*P* < 0.05为差异有统计学意义，*P*值均采用双侧检验。

## 2 结果

### 2.1 一般情况

在273例EGFR敏感突变的晚期NSCLC患者中，23例（8.42%）合并中等到大量MPCE患者纳入分析。中位年龄55岁，女性15例，不吸烟18例。23例患者的一般情况见表 1。

**1:** 患者的一般情况 Patient characteristics (*n*=23)

Characteristics	Data
Age (yr)	
Median (range)	55 (29-81)
Gender	
Male	8
Female	15
Smoking history	
Current or ever	5
Never	18
ECOG PS	
0-1	3
2	15
3-4	5
Histology	
Adenocarcinoma	16
Non-adenocarcinoma	7
Type of *EGFR* gene mutations	
18	2
19	9
21	12
ECOG PS: Eastern Cooperative Oncology Group performance status. EGFR: epidermal growth factor receptor.	

### 2.2 疗效及预后

#### 2.2.1 恶性心包积液

23例患者经过治疗，有5例获得CR，13例PR。截至2016年12月31日随访结束，23例患者中18例（78.2%）进展，5例（21.7%）未进展，中位心包内PFS为247天（95%CI: 129-364）。分析显示，患者的临床特征，包括性别、年龄分组（< 55岁与≥55岁）与心包内PFS无相关性，差异均无统计学意义（*P* > 0.05）。因PS评分、吸烟状态、病理类型、分期及*EGFR*突变位点等各亚组例数不均衡，未做统计学处理（表 2）。

**2:** 患者心包内PFS Pericardium PFS for patients

	*n*	Events	Median PFS (d)	95%CI (d)	*P*
Gender					0.758
Male	8	4	222	77-367	
Female	15	14	260	85-435	
Age (yr)					0.978
< 55	9	5	249	0-526	
≥55	14	13	246	124-367	
Local therapy (+gefitinib)					0.032
Yes	9	7	460	209-711	
No	6	3	131	58-203	
Gefitinib therapy (+HCPT)					0.008
Yes	15	10	460	209-711	
No	8	8	94	11-177	
PFS: progression-free survival.					

吉非替尼/HCPT组患者PFS（460天，95%CI: 209-711）优于化疗/ HCPT组患者（94天，95%CI: 11-177；*P*=0.008）（图 1）；吉非替尼/HCPT组患者的PFS优于吉非替尼单药组患者（131天，95%CI: 58-203；*P*=0.032）（图 2），差别均有统计学意义。上述结果显示吉非替尼/HCPT组较吉非替尼单药组及化疗/HCPT组在延长心包PFS方面更具优势。

**1:**
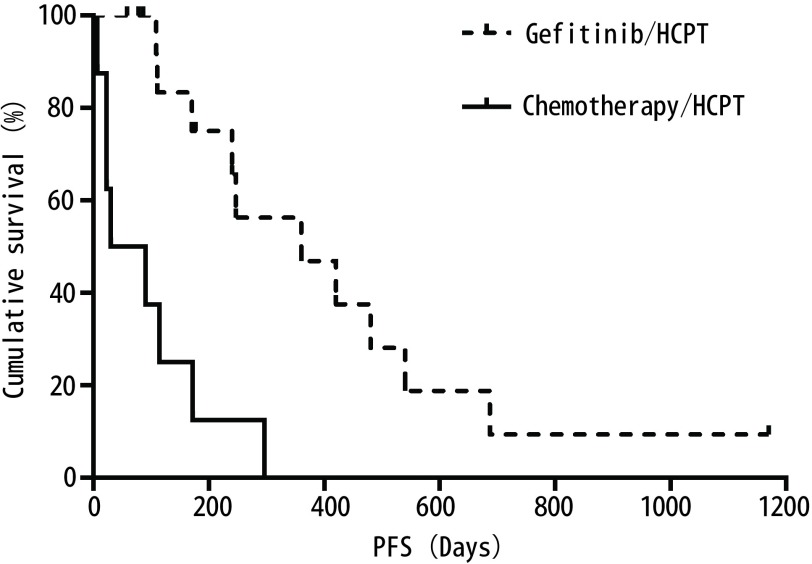
一线接受心包灌注HCPT联合吉非替尼或静脉化疗患者的心包内PFS Pericardium PFS for patients according to first-line gefitinib/HCPT or chemotherapy/HCPT therapy. HCPT: hydroxycamptotheci; PFS: progression free time.

**2:**
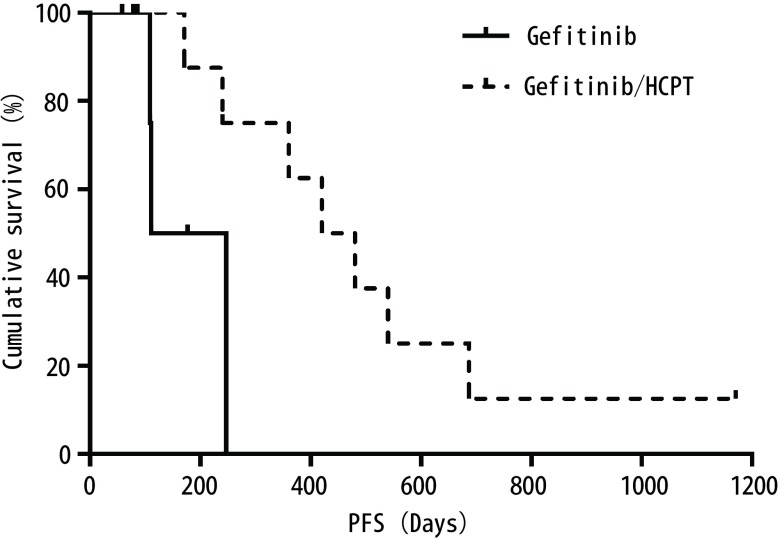
一线接受吉非替尼是否联合心包灌注HCPT局部治疗患者的心包内PFS Pericardium PFS for patients according to first-line gefitinib/HCPT or gefitinib therapy

#### 2.2.2 实体瘤

对23例入组患者进行肺部原发灶疗效的评价，1例CR，5例PR，12例SD，5例PD。吉非替尼/HCPT组患者疗效优于化疗/HCPT组患者（DCR：86.7% *vs* 62.5%；ORR：33.3% *vs* 12.5%）。吉非替尼/HCPT组与吉非替尼单药组的患者，肺部肿瘤疗效ORR无差异：33.3%（3/9）*vs* 33.3%（2/6）；DCR稍增加：88.9%（8/9）*vs* 83.3%（5/6）。

### 2.3 不良反应

研究中没有观察到治疗相关的死亡、过敏反应以及肝和肾功能障碍。吉非替尼/HCPT组及吉非替尼单药组的主要毒性为皮疹（20.0%, 3/15）。而化疗/HCPT组的不良反应主要包括Ⅲ级/Ⅳ级白细胞减少症（37.5%, 3/8）、贫血（12.5%, 1/8）和血小板减少症（37.5%, 3/8）。化疗/HCPT组其他的毒性主要是恶心呕吐（37.5%, 3/8）。

## 3 讨论

吉非替尼是对*EGFR*敏感突变的NSCLC患者的有效治疗方法。First-Signal^[20]^研究中亚洲未接受过化疗、非吸烟的肺腺癌患者一线使用TKI治疗，中位PFS为6.1个月，OS达21.3个月。西班牙的一项回顾性研究350例*EGFR*敏感突变且使用EGFR-TKI治疗的肺癌患者，ORR为71%，中位PFS 14个月，中位OS达到27个月^[21]^。本研究对入组患者的临床资料进行回顾性分析，中位心包内PFS为247天。一线接受吉非替尼治疗的患者PFS优于接受化疗的患者（*P*=0.008）。台湾Wu等^[22]^研究发现伴发MPCE的肺腺癌使用TKI治疗的患者中位OS明显长于未使用者（16.8个月*vs* 6.8个月），也证实了吉非替尼对全身疾病和恶性心包积液的控制均优于化疗。

本研究共入组23例为*EGFR*敏感突变的晚期NSCLC患者初诊合并MPCE，临床特征分析显示中位年龄55岁，多见于腺癌、不吸烟患者。273例晚期*EGFR*敏感突变的NSCLC患者中，23例含有中到大量MPCE，心包转移发生率为8.42%。既往研究表明恶性心包积液绝大多数由恶性肿瘤心包转移引起，尸检结果显示肿瘤患者心脏的心包受侵率为5%-12%^[23]^，这与我们此次的研究结果相符。同时我们研究发现一线吉非替尼联合局部心包灌注羟基喜树碱的中位心包内PFS为460天，而单纯吉非替尼治疗的PFS仅为131天，两者有统计学差异（*P*=0.032）。心包积液的治疗可分为全身治疗和局部治疗两种。前者适用于化疗效果好的疾病，如小细胞肺癌、恶性淋巴瘤等，而全身化疗对恶性心包积液疗效只有34.5%^[24]^，因此大量心包积液，必须做局部治疗。目前，穿刺引流、心包腔内给药及处理原发肿瘤均为恶性心包积液的主要治疗手段，而单纯引流积液不能有效控制心包积液，因此需积极抗肿瘤治疗^[8]^。Martinoni等^[9]^认为，心包腔灌注药物的局部治疗可以达到70%-90%的有效率。以往的研究表明，硫酸博莱霉素、四环素、顺铂、丝裂霉素C和卡铂能有效的控制恶性心包积液^[7, 25-29]^，但这些药物往往会导致严重的消化道反应、心肌损伤和心律失常，致使患者无法耐受。因此我们迫切的需要寻找高效低毒的心包灌注药物。HCPT是喜树碱的衍生物，它是从中国的喜树中被分离出来的化合物。HCPT是一种拓扑异构酶抑制剂，能够干扰DNA的复制，从而发挥其抗肿瘤的作用^[30]^。既往研究表明羟基喜树碱常用于恶性浆膜腔积液的治疗，疗效较好且不良反应轻微^[14, 15]^。本研究中羟基喜树碱心包灌注后未见明显不良反应，其中年龄最大的一例患者为81岁，应用羟基喜树碱后也未见明显不良反应，提示对年老体弱和不能耐受强烈化疗药物的晚期患者，羟基喜树碱不失为局部治疗恶性心包积液的良好药物，值得临床上进一步应用。

与此同时，我们对患者的肺部原发灶疗效也进行了评估，吉非替尼/HCPT组患者疗效优于化疗/HCPT组的患者，且不良反应少，该结果与局部MPCE控制情况一致。吉非替尼/HCPT组患者与吉非替尼单药组患者肺部原发灶的疗效无明显差别，该结果提示局部治疗作为MPCE重要的控制手段，如何与全身治疗配合，从而增加患者的生存获益是目前临床医生面临的重要挑战。

综上所述，吉非替尼联合心包灌注HCPT治疗可使EGFR突变合并MPCE的晚期NSCLC患者获得良好的心包内疾病控制。本文的不足在于例数偏少，治疗不统一，尚不能做OS分析，我们将对全组患者继续随访。为探索最佳的治疗模式，今后有必要开展多中心的前瞻性对照研究，以靶向治疗为基础，权衡靶向治疗与局部治疗的合理顺序，做好患者的全程管理，使得患者生存获益最大化。
